# Capacity Sharing and Capacity Investment of Environment-Friendly Manufacturing: Strategy Selection and Performance Analysis

**DOI:** 10.3390/ijerph17165790

**Published:** 2020-08-10

**Authors:** Lei Xie, Hongshuai Han

**Affiliations:** 1 School of Management, Shandong University, Jinan 250100, China; lxie@sdu.edu.cn; 2 College of Management and Economics, Tianjin University, Tianjin 300072, China

**Keywords:** capacity sharing, capacity investment, environment-friendly manufacturing, co-opetitive game, stackelberg game

## Abstract

Many small manufacturing factories suffer insufficient environment-friendly capacity after eliminating the outdated and environmental-harmful production capacity according to stringent environmental rules and regulations. This paper analyzes two strategies that the manufacturer with limited environment-friendly capacity may take to tackle this problem, i.e., investing in building environment-friendly capacities and collaborating with the manufacturer with sufficient environment-friendly capacity in capacity sharing. In a supply chain with two competing manufacturers, this paper builds game-theoretical models and investigates equilibrium solutions under three scenarios (no capacity investment or sharing, capacity investment, and capacity sharing). Then this research investigates the feasible regions of these two strategies and compares the performance of each manufacturer under each scenario. The findings show that both capacity investment and capacity sharing can effectively reduce the profit loss of the manufacturer with limited capacity, while only capacity sharing benefits both manufacturers. The feasibility of these two strategies depends on the initial capacity volume and the capacity investment cost coefficient of the manufacturer with limited capacity. Moreover, the preference of the manufacturer with limited capacity for each strategy depends on the capacity investment cost coefficient. When the capacity investment cost coefficient is relatively high, the win-win situation exists for supply chain members. Furthermore, with the use of chaos theory, the paper shows how to adjust the capacity investment in each period to keep the system stable.

## 1. Introduction

Increasing environmental awareness and strict environmental regulations posit significant challenges to the manufacturing industry. The Sustainable Development Goals (2030 UN Agenda) highlights the importance of ensuring responsible production to use resources and protect the environment efficiently. In China, the government launched a robust environmental protection campaign in 2017, resulting in a dilemma for several small manufacturing factories that lacked pollutant treatment facilities. For example, in June 2018, the State Council of China issued the “Three-year Action Plan to Win the Blue Sky Defence War.” A significant number of “scattering and polluting” manufacturing enterprises had shut down, and many manufacturers are required to phase out the outdated and environment-damaging production capacity, as well as rectify and upgrade their manufacturing process to be environmental-friendly. To meet stiffening environmental regulations, an increasing number of manufacturers have invested in purchasing environmental-friendly equipment, promoting technological improvement, upgrading manufacturing capacity to improve resource utilization and reduce emissions.

This research is motivated by the establishment of a air-conditioning shared factory in Dezhou, China. Under the environmental protection campaign, the owner of this factory, i.e., Zhongwei Group, is a strong local enterprise which took the opportunity of capacity expansion and upgrading, and invested heavily to import several sets of the environmental-friendly production line to successfully passed the national assessment. Considering a category of its environment-friendly equipment that is essential to the production process runs only 1–2 h per day and several green production lines may be idle in a certain period, this enterprise proposed the pattern of “shared factory” to utilize its production capacity and earn more profits fully. The shared factory offers manufacturers with limited manufacturing capacity and resources to acquire manufacturing services. It has shared the environment-friendly equipment and green production lines with several local air-conditioning firms that cause noise and air pollution in the production process.

For many small and medium manufacturers that cause pollution in their production processes, investment in environment-friendly equipment and production line may cause a high potential risk since they have comparatively small order volumes and tend to experience severe financial burden. Therefore, the enterprise should make a prudent investment decision and it is critical to investigate the influence of environment-friendly capacity investment on the enterprise’s performance. The mode of capacity sharing offers an alternative for manufacturers with limited environment-friendly capacity to utilize the green production line. Taking advantage of capacity sharing, the manufacturers with insufficient capacity can continue to schedule their production planning by purchasing manufacturing service from manufacturers with sufficient environment-friendly capacity without excessive investment. The capacity sharing through shared factory have achieved great success in the capacity allocation and environmental protection. For example, in Wucheng County, Dezhou, China, although 1017 polluting companies shut down under the environmental supervision, 96 shared factories were established with the support of the government to help the small manufacturers revive. It is reported that the order volume of manufacturers rose an average of 40% after joining in one shared factory (https://www.sohu.com/a/230705467_100023701). Taking advantage of the environmental renovation and manufacturing mode innovation, the average concentrations of PM_2.5_, PM_10_, SO${}_{2}$, and NO${}_{2}$ in Wucheng County in 2017 decreased by 19.1%, 17.1%, 11.4%, and 10% year-on-year, respectively (https://baijiahao.baidu.com/s?id=1604960902183948890&wfr=spider&for=pc). However, capacity sharing is highly flexible and dependent on the availability of the manufacturers’ idle capacity. It is interesting to explore the conditions when the capacity sharing is conducive to the mutual benefits of both the capacity provider and the capacity requester.

Therefore, it is critical to investigate how the investment or sharing of environment-friendly capacity affect the manufacturers’ performance outcomes. This paper focuses on the manufacturers’ choice between the capacity investment and capacity sharing and examines the influence of each strategy on each manufacturer’s performance. Based on the case of the air-conditioning shared factory we mentioned before, we consider the demand side and the supply side of environment-friendly capacity are competitors who produce similar products. Thus we will examine each manufacturer’s preference for capacity investment and capacity sharing with the existence of competition among them. Specifically, the research questions are as follows.

(1) How to choose between capacity investment and capacity sharing?
(2) What are the influences of capacity investment and capacity sharing on each manufacturer’s performance?
(3) How to adjust the capacity investment to keep the decision-making system stable?

To solve these problems, we first derive the equilibrium results under different scenarios, i.e., no capacity investment or sharing, capacity investment, and capacity sharing with the use of game theory. Then we analyze the application scopes of these two strategies to show their feasibility. We further study each manufacturer’s preference for these two strategies by making a comparison of the profitabilities. Moreover, given the high sunk costs of investment in environment-friendly capacity, manufacturers are cautious about making the capacity investment. Therefore, by using the chaos theory, this paper considers that the manufacturer’s investment in capacity is gradually adjusted, and introduces bifurcation diagrams, time series of decisions and chaotic attractors, to study the nonlinear characteristics and put forward the conditions of keeping the system stable.

Theoretically, this paper enriches the literature on both environmental management and capacity management in manufacturing by comparing the effectiveness of environment-friendly capacity investment strategy and the environment-friendly capacity sharing strategy and by modeling a co-opetition game between two competing manufacturers. Besides, this paper explores the conditions for maintaining system stability in the pursuit of profitability, which throw lights on the dynamic supply chain management. In practice, this paper provides managerial insights for a limited-capacity manufacturer on approaches towards green manufacturing and offers decision support to achieve the win-win situation between two competing manufacturers. Furthermore, this paper assists the limited-capacity manufacturer in adjusting the investment on the environment-friendly capacity to balance the profitability and system stability.

The remainder of this paper is organized as follows. Section 2 reviews the studies closely related to our research. Section 3 describes the main models and corresponding equilibrium outcomes. The comparisons on application scopes of these two strategies and the profitabilities of manufacturers are made in Section 4. The stability analysis is provided in Section 5. Section 6 provides managerial insights for both two manufacturers and the government. Section 7 concludes this paper and suggests future research directions. All the equilibrium profits, proofs and explanations are deferred to the Appendix A, Appendix B and Appendix C for clarity. Specifically, the equilibrium profits are given in Appendix A; the proofs for Lemmas and Propositions are given in Appendix B; the explanation of data selection and calculation is given in Appendix C.

## 2. Literature Review

This investigation is part of a wider literature about environmental management and firm value in China and elsewhere. The research is closely related to three streams of literature. The first stream focuses on environment-friendly manufacturing. The second stream concentrates on capacity investment and capacity sharing strategies, and the last stream is related to the stability analysis of supply chain systems.

### 2.1. Environment-Friendly Manufacturing

Environment-friendly manufacturing can be achieved by updating the manufacturing technique and adopting the environment-friendly production line. Some researchers pay attention to the relationship between environmental management and firm performance. For example, Lu et al. [1] shows that environmental management has no significant effect on firm value, and the prior financial performance does not affect the level of environmental management. Many researchers focus on the measures taken to improve the degree of environment-friendly in the manufacturing process. To cite a few, Liu et al. [2] propose an environment-friendly manufacturing process by taking high-speed dry milling as the final manufacturing process. Jiang et al. [3] establish an efficient and environment-friendly limestone calcination process with ${\mathrm{CO}}_{2}$ looping and recovery to solve the associated environmental issue. Menor et al. [4] specify the different characteristics of cork waste depending on the stage of the process they are generated in, finding that the use as lightweight aggregate can be considered as an environmentally friendly use.

The closed-loop supply chain is beneficial to the environment [5]. In order to improve environmental performance, some companies design products for recycling. Recovering the used products with reverse logistics, companies make re-manufactured products and sell them to the specific customers, forming a closed-loop supply chain. Chang et al. [6] investigate the joint tax-subsidy mechanism in an environment-friendly supply chain consisting of a government, a manufacturer, and a recycler. It shows that such a joint tax-subsidy mechanism can motivate the manufacturer to pursue eco-innovation. Considering consumers’ bargaining, Zhu et al. [7] study a closed-loop supply chain of waste electrical and electronic equipment, showing that the retailers and recycling platforms will reduce the recovery prices, and remanufacturers will improve the transfer payment price to improve their profits.

Although most above-mentioned researches highlight the way towards environment-friendly manufacturing, they did not incorporate the sharing of environment-friendly manufacturing capacity. In this paper, we focus on a supply chain with competing manufacturers in which one has limited environment-friendly capacity and the other with surplus environment-friendly capacity. We compare two ways for the manufacturer to address the capacity limitation, i.e., capacity investment and capacity sharing.

### 2.2. Capacity Investment and Capacity Sharing Strategies

In this paper, we focus on the manufacturing firms’ choices between capacity investment and capacity-sharing strategies. Capacity investment is a traditional way widely used to address capacity limitations. Chen and Chen [8] consider a manufacturer that serves a set of retail stores determines capacity at the beginning of the planning horizon and makes a capacity investment decision. It provides a mechanism to compute fair cost allocation. Hach et al. [9] develop a dynamic capacity investment model to assess the effects of different capacity market design options in three scenarios: (1) no capacity market, (2) a capacity market for new capacity only, and (3) a capacity market for new and existing capacity. They compare the results according to three key dimensions of electricity policy—affordability, reliability, and sustainability. Berling and Englarsson [10] investigate a service provider’s optimal investment in service capacity, and its environmental implications under a volume contract and a capacity contract, respectively. Yang et al. [11] emphasize the high risk in capacity investment. With two capacity sharing contracts introduced, they investigate the role of a retailer in a manufacturer’s capacity investment strategies. Jain and Hazra [12] model a supply chain with two upstream suppliers competing on capacity investment to fulfill a buyer’s requirement, showing that an increase in the variability of suppliers’ capacity will decrease the suppliers’ investments.

Capacity sharing, as an effective way to solve the capacity limitation problem, had attracted great attention to both academia and practitioners in recent years. Yang and Anderson [13] formulate a game-theoretical model where each firm has an existing capacity and faces both fixed and variable costs in purchasing additional capacity. They compare the outcomes obtained in the scenarios where the firms simultaneously (or sequentially) make their expansion decisions, and then simultaneously decide their production decisions. Seok and Nof [14] propose the capacity sharing model among independent and non-competitive manufacturers. Recently, more researchers focus on the capacity sharing between competing firms. Guo and Wu [15] investigate optimal strategies and firm profitability considering capacity sharing between competing firms under two scenarios, i.e., the capacity-sharing price is determined before/after price setting in the buyer market. Li and Zhang [16] study the cooperation game between two shipping forwarders who share shipping capacities, finding that the capacity reservation between competing forwarders benefits both the carrier and the forwarders.

Since capacity sharing or investment can successfully address the capacity limitation, it is vital to analyze their effectiveness. From the perspective of the capacity seller, Yu et al. [17] explore the firms’ choices of either operating their own production/service facilities or operating a shared facility. Qi et al. [18] analyze a firm’s optimal strategy to adjust its capacity by comparing two scenarios in which the capacity adjustment cost increases significantly or remains unchanged concerning the number of adjustments. To differ from them, we explore the strategic choices between capacity investment and capacity sharing for a manufacturer with limited environment-friendly capacity, and further investigate the adjustment in capacity investment in order to keep the system stable and optimal.

### 2.3. Application of Chaos Theory in the Supply Chain

We use chaos theory to study the stability analysis of the capacity investment decisions. Chaos theory is well known in the study of the meteorological system. Hwarng and Xie [19] reveal the phenomenon of chaos in the supply chain system. Chaos may occur in the supply chain financial system. Ma and Li [20] examine the nonlinear characteristic of a financial supply chain system and provide a method to control the chaotic system. Chen and Wang [21] establish a three-dimensional fractional calculus game model in a financial system of blockchain supply chain and study the chaos phenomenon in such a supply chain. Currently, an increasing number of researches focus on the chaos in the dual-channel supply chain [22,23,24,25], closed-loop supply chain [26,27,28]. As chaos phenomenon is usually harmful, Goksu et al. [29] study the chaos control for a supply chain. Few studies focus on the complexity analysis of capacity investment in the supply chain. In this paper, we consider the manufacturer with limited environment-friendly capacity to be bounded rational, which adjust its capacity investment in each period. In such a nonlinear dynamic supply chain system, we study its stability performance and provide managerial insights for these firms to keep the system stable.

## 3. The Model

In this paper, we consider two competing manufacturers: one (M1) is with limited environmental-friendly capacity, and another (M2) is with sufficient environmental-friendly capacity. They make a quantity competition, which makes sense in the productions with long production cycles. The subscript i=1,2 represents the parameters or variables belonging to M1 and M2, respectively. The inverse demand function is given as $p_{i}=a-q_{i}-bq_{j}$, where j=3−i. $p_{i}$ is the selling price of the product *i*. $q_{i}$ and $q_{j}$ represent the selling quantities of product *i* and its competitive product *j*. *a* is the potential market size. For ease of analysis, we normalize a=1. *b* satisfying 0≤b≤1 represents the substitution relationship between the two products. If b=0, there is no substitution relationship. If b=1, two products can perfectly substitute each other. In this paper, we consider a fierce competition between the two manufacturers. Hence, *b* is assumed to be relatively large, even equal to 1. To address the capacity limitation, M1 can choose from capacity investment or capacity sharing. Therefore, we consider three scenarios: no capacity investment or sharing case (with parameters and variables indexed by superscript NN), the capacity investment case (with parameters and variables indexed by superscript IN) and the capacity sharing case (with parameters and variables indexed by superscript NS).

For both the capacity buyer and seller, their cost models consist of two parts, i.e., variable cost and fixed cost. The fixed cost, which is not influenced by the production quantity, such as the equipment setup cost and the cost of energy and laborers. The variable cost related to the production quantity, such as the machining cost and the raw materials cost. Since the fixed costs exert no essential influence on the equilibrium analysis, we normalize the fixed costs of both the capacity buyer and the seller to be zero. Considering the competing manufacturers produce similar products, to simplify and without loss of generality, we assume they have the same variable production cost which is denoted as *c*. In the scenario of environmental-friendly capacity sharing, M2 charges manufacturing service fee *w* per unit product. For M1, the cost of sharing contains the fixed costs, which include initial searching cost and bargaining cost, and the supervision cost in the capacity sharing transaction, as well as the variable cost, i.e., the manufacturing service cost which equals to the unit manufacturing service fee times product quantity. Similarly, due to the fixed costs has no essential impact on the equilibrium results, we normalize them to zero and focus on the optimal pricing of unit manufacturing service fee and its effect on the sharing decision.

### 3.1. No Capacity Investment or Sharing Case

In this section, we consider that no strategies is adopted to address the capacity limitation. M1 and M2’s problems are:

(1) $$\max\pi_{1}=\left(p_{1}-c\right)q_{1},\;\;s.t.,\;q_{1}\leq k$$

(2) $$\max\pi_{2}=\left(p_{2}-c\right)q_{2}$$

Although M1’s capacity may be large, only a part of it is environment-friendly and meet the requirement of the local government. *k* represents the environment-friendly capacity of M1, and the condition $q_{1}\leq k$ means that only environment-friendly manufacturing process is permitted. Using superscript BM to index the variables in benchmark case, where both M1 and M2 are with sufficient capacities, we have:

**Lemma** **1.** (1) If both M1 and M2 are with sufficient environment-friendly capacities, the optimal quantities $q_{i}^{BM*}=\frac{1-c}{2+b}$, the profits are $\pi_{i}^{BM*}=\frac{{\left(c-1\right)}^{2}}{{\left(b+2\right)}^{2}}$; (2) If M1 is with insufficient environment-friendly capacity and does not invest capacity or borrow capacity from M2, the equilibrium outcomes are $q_{1}^{NN*}=k$, $q_{2}^{NN*}=\frac{1}{2}\left(1-c-bk\right)$, and the optimal profits are $\pi_{1}^{NN*}=\frac{k}{2}\left(b^{2}k+b\left(c-1\right)-2\left(c+k-1\right)\right)$, $\pi_{2}^{NN*}=\frac{1}{4}{\left(bk+c-1\right)}^{2}$.

All the proofs for Lemmas and Propositions are given in Appendix B. If both manufacturers have sufficient environmental-friendly capacities, neither of them needs to invest capacity or borrow capacity from each other. Then the constraint $q_{i}\leq k$ is relaxed and the profit function is given as $\pi_{i}=\left(p_{i}-c\right)q_{i}$. The optimal quantities $q_{i}^{BM*}=\frac{1-c}{2+b}$. 0≤c<1 should be satisfied as the basic condition throughout this paper to ensure non-negative equilibrium results. Hence, in the following paper, when we consider the insufficient environmental-friendly capacity case, the capacity of M1 satisfies $k<k^{\prime }$, where $k^{\prime }=\frac{1-c}{2+b}$.

As $k<k^{\prime }$ is the sufficient condition of the following conditions $k<\frac{1-c}{b}$, $k<-\frac{\left(b-2)(c-1\right)}{b^{2}-2}$, $k<-\frac{\left(b-2)(c-1\right)}{2(b^{2}-2)}$, $\frac{d\pi_{1}^{NN*}}{dk}=b^{2}k+\frac{1}{2}b\left(c-1\right)-c-2k+1>0$ and $\frac{d\pi_{2}^{NN*}}{dk}=\frac{b}{2}\left(bk+c-1\right)<0$, we can infer that $q_{2}^{NN*}>0$, $\pi_{1}^{NN*}>0$, $\pi_{1}^{NN*}$ increases in *k* and $\pi_{2}^{NN*}$ decreases in *k*, which implies that the limitation in one’s capacity hurts its profit but benefits its rival’s.

**Proposition 1.**  (1) The limitation in environment-friendly capacity hurts M1’s profit, but contributes to M2’s profit, i.e., $\pi_{1}^{NN*}<\pi_{1}^{BM*}$, $\pi_{2}^{NN*}>\pi_{2}^{BM*}$. (2) M1 should make full use of the existing limited environment-friendly capacity to maximize the profit.

It shows that the limitation in environmental-friendly capacity is not only harmful to the environment but also detrimental to M1’s profit. Although the buyer will raise the retail price when the capacity is limited, the lack of production quantity hurts its profit. This also implies that the buyer can get more profits if it can borrow some capacity from others or invest capacities itself. $\pi_{1}^{BM*}=\frac{{\left(c-1\right)}^{2}}{{\left(b+2\right)}^{2}}$ is the highest profit M1 can get even when the capacity *k* is far greater than the optimal $k^{\prime }$. However, if $k<k^{\prime }$, the profits will be lower than $\pi_{1}^{BM*}$. Based on Lemma 1, we can infer that the best choice for the limited-capacity manufacturer is to make full use of the existing environmental-friendly capacity.

### 3.2. The Capacity Investment Case

In this section, we consider the case that M1 invests the new environmental-friendly capacities in order to meet the demand of the customers and the requirement of the government. We use a linear cost function $c_{k}=r\Delta k$ for capacity investment, which is widely used in the existing literature Boonman et al. [30], Goyal and Netessine [31]. In such a cost function, the unit cost of capacity is *r* Xiao et al. [32]. The expansion capacity is Δk. Making full use of the existing capacity *k*, the sales of M1 will be $q_{1}=k+\Delta k$ and the prices are:

(3) $$p_{1}=1-\left(k+\Delta k\right)-bq_{2}$$

(4) $$p_{2}=1-b\left(k+\Delta k\right)-q_{2}$$

The profit functions of these two manufacturers are given as:

(5) $$\pi_{1}=\left(p_{1}-c\right)\left(k+\Delta k\right)-r\Delta k$$

(6) $$\pi_{2}=\left(p_{2}-c\right)q_{2}$$

The game sequence is that: M1 first determines the capacity investment quantity Δk and then M2 decides the quantity $q_{2}$. With backward induction, we need to solve out the best response function (BRF) of $q_{2}$ in the second stage. Based on Proposition 1, the BRF of $q_{1}$ is $q_{1}=k+\Delta k$. The BRF of $q_{2}$ can be solved out by the first order condition, which is given as $q_{2}=\frac{1}{2}\left(-b\left(\Delta k+k\right)-c+1\right)$ after checking $\frac{d^{2}\pi_{2}}{d{q_{2}}^{2}}=-2<0$. Substituting the BRFs into the profit function (6), we can get the optimal capacity investment quantity $\Delta k^{IN*}$ after checking $\frac{d^{2}\pi_{1}}{d{\Delta k}^{2}}=-2+b^{2}<0$.

**Lemma** **2.** The equilibrium outcomes in scenario IN are $\Delta k^{IN*}=\frac{-2b^{2}k-bc+b+2\left(c+2k+r-1\right)}{2(b^{2}-2)}$, $q_{1}^{IN*}=\frac{b(-c)+b+2(c+r-1)}{2(b^{2}-2)}$, $q_{2}^{IN*}=\frac{b^{2}\left(c-1\right)+2b\left(c+r-1\right)-4c+4}{4(2-b^{2})}$.

The equilibrium profits $\pi_{1}^{IN*}$ and $\pi_{2}^{IN*}$ are given in Appendix A. It is reasonable that the sales cannot be larger than that in sufficient capacity case, i.e., $0<\Delta k\leq q_{1}^{BM*}-k$. If $\Delta k>q_{1}^{BM*}-k$, the overinvestment $\Delta k-(q_{1}^{BM*}-k)$ will reduce the profits of M1 because the highest profit can be obtained when the quantity is $q_{1}^{BM*}$. The capacity investment should just cover the shortfall in capacity, i.e., $\Delta k=(q_{1}^{BM*}-k)$.

When $r>\frac{b^{2}\left(1-c\right)}{2(b+2)}$, we have $k+\Delta k<q_{1}^{BM*}$. Thereby it is inferred that when the unit cost of capacity is relatively low, i.e., $r<\frac{b^{2}\left(1-c\right)}{2(b+2)}$, we get $\Delta k>q_{1}^{BM*}-k$. As we mentioned above, the overinvestment is detrimental to M1’s profit, then the optimal capacity investment is $q_{1}^{BM*}-k$ and the sales is $q_{1}^{BM*}$.

**Proposition** **2.** *Only when the environment-friendly capacity is fiercely limited, i.e., $k<k^{IN}$, will M1 invest environment-friendly capacity, where $k^{IN}=\frac{(b-2)(1-c)+2r}{2(b^{2}-2)}$.*

Observing Δk>0 when $k<k^{IN}$, we can infer that only when the initial environmental-friendly capacity *k* is sufficiently low will M1 invest capacity. Otherwise, the optimal investment in capacity is zero because optimal investment decreases in the cost coefficient *r* and the capacity *k*. If *k* is sufficiently large, the optimal investment will decrease to zero. Proposition 2 indicates that not all the limited-capacity buyers can reach the equilibrium when investing in the environmental-friendly capacities. This does not mean that the buyer will lose from investing capacities. Instead, it means that the seller may not accept the buyer’s decisions or requirements. As $0<c<\frac{-2+b+2r}{-2+b}$ guarantees $k^{IN}>0$. If *c* locates outside this range, no one will consider to invest capacity. Therefore, we assume the unit manufacturing cost is sufficiently low, i.e., $0<c<\frac{-2+b+2r}{-2+b}$ to avoid the meaningless case.

As for the unit cost *r* of capacity investment, note that $\underline{r}=-\frac{b^{2}\left(c-1\right)}{2(b+2)}$, $\bar{r}=b^{2}k+\frac{1}{2}b\left(c-1\right)-c-2k+1$, only when the unit cost *r* satisfies $\underline{r}<r<\bar{r}$ will M1 invest capacity. $\bar{r}-\underline{r}=\frac{\left(b^{2}-2\right)\left(\left(b+2\right)k+c-1\right)}{b+2}$. Obviously, when $k<k^{\prime }$, $\frac{\left(b^{2}-2\right)\left(\left(b+2\right)k+c-1\right)}{b+2}>0$ and the range $\left(\frac{b^{2}\left(1-c\right)}{2(b+2)},b^{2}k+\frac{1}{2}b\left(c-1\right)-c-2k+1\right)$ of *r* exists. $\underline{r}<r$ guarantees $k+\Delta k<k^{\prime }$, $r<\bar{r}$ guarantees Δk>0. If $r<\underline{r}$, the optimal investment $\Delta k=q_{1}^{BM*}-k$.

As $\frac{\partial k^{IN}}{\partial c}=\frac{2-b}{2b^{2}-4}<0$, $\frac{\partial k^{IN}}{\partial r}=\frac{1}{b^{2}-2}<0$, we can infer that the scope of application for capacity investment strategy will decrease with the increases of *c* and *r*. The increase of the production cost, as well as the increase of the capacity investment cost, will prevent the manufacturers from investing the environment-friendly capacities.

**Proposition 3.**  (1) Capacity investment can effectively reduce the profit loss caused by insufficient environment-friendly capacity. The loss cannot be made up by investing environment-friendly capacity, i.e., $\pi_{1}^{BM*}>\pi_{1}^{IN*}>\pi_{1}^{NN*}$. (2) On the contrary, M2 can always benefit from its rival’s capacity limitation. If M1 invests environment-friendly capacities, M2 will get less profits, i.e., $\pi_{2}^{BM*}<\pi_{2}^{IN*}<\pi_{2}^{NN*}$.

M1 can maximize profits $\pi_{1}^{IN*}$ when it has sufficient environmental-friendly capacity with the sales equaling to $q_{1}^{BM*}$. For a limited-capacity M1, it can get sufficient capacity and then increase the profit by investing capacity. However, the cost of capacity investment determines that the profit $\pi_{1}^{IN*}$ can never reach $\pi_{1}^{BM*}$. In other words, the capacity investment can only reduce the profit loss caused by insufficient production capacity; however, the profitability under case IN can not reach that under the benchmark case. Even so, the investment in capacity benefits the buyer M1. For the sufficient-capacity M2, M1’s capacity investment will hurt M2’s profit as M1 can provide more products competing with M2 in the market.

### 3.3. The Capacity Sharing Case

If M1 is not willing to take the risk of capacity investment, it can borrow the environmental-friendly equipment from M2. In this section, we consider M1 to “buy” capacity from M2 to replenish capacity, i.e., to use M2’s environmental-friendly equipment to produce. It determines the optimal quantity bought from M2 to maximize the profit after receiving the quoted unit manufacturing service fee *w* set by M2. The sequence of events in this scenario is that the capacity seller M2 quotes manufacturing service fee *w* for per-unit shared capacity, then M1 decides the capacity $q_{t}$ bought from M2 and M2 decides its quantity $q_{2}$ simultaneously.

In this scenario, the retail prices are modeled as:

(7) $$p_{1}=1-\left(q_{t}+k\right)-bq_{2},$$

(8) $$p_{2}=1-b\left(q_{t}+k\right)-q_{2}.$$

The profit functions in this scenario are given as:

(9) $$\pi_{1}=\left(p_{1}-c\right)k+\left(p-w\right)q_{t},$$

(10) $$\pi_{2}=\left(p_{2}-c\right)q_{2}+\left(w-c\right)q_{t}.$$

We can get the BRFs $q_{t}=\frac{b^{2}\left(-k\right)-bc+b+4k+2w-2}{b^{2}-4}$, $q_{2}=\frac{b(-w)+b+2c-2}{b^{2}-4}$ after checking $\frac{d^{2}\pi_{1}}{d{q_{t}}^{2}}=-2<0$, $\frac{d^{2}\pi_{2}}{d{q_{2}}^{2}}=-2<0$. Substituting these BRFs into $\pi_{2}$, we have $\frac{d^{2}\pi_{2}}{dw^{2}}=\frac{4}{b^{2}-4}<0$ and the unique optimal solution $w^{NS*}$ can be obtained.

**Lemma** **3.** When M1 buys capacity from M2, the equilibrium outcomes are $w^{NS*}=\frac{b^{4}k+b^{3}\left(c-1\right)+2b^{2}\left(c-4k+2\right)-8\left(c-2k+1\right)}{6b^{2}-16}$, $q_{t}^{NS*}=\frac{2(b^{2}\left(-k\right)-bc+b+c+2k-1)}{3b^{2}-8}$, $q_{2}^{NS*}=\frac{\left(b-2\right)(b^{2}k+b\left(c+2k-1\right)+4\left(c-1\right))}{16-6b^{2}}$.

**Proposition** **4.** *Only when the environment-friendly capacity is fiercely limited, i.e., $k<k^{NS}$, will M1 buy environment-friendly capacity from M2, where $k^{NS}=\frac{\left(b-1)(1-c\right)}{b^{2}-2}$.*

The equilibrium profits $\pi_{1}^{NS*}$ and $\pi_{2}^{NS*}$ are given in Appendix A. To ensure $q_{t}^{NS*}>0$, we have the condition $0<k<k^{NS}$. It is not difficult to prove that $0<k<k^{\prime }$ is the sufficient condition of $q_{t}^{NS*}+k<q_{1}^{BM*}$. Hence, $0<k<k^{NS}$ ensures that the total quantities including its own capacity *k* and the order $q_{t}^{NS*}$ are not larger than the optimal quantity in sufficient capacity case, i.e., $k<k+q_{t}^{NS*}<q_{1}^{BM*}$.

As $\frac{\partial k^{NS}}{\partial b}=\frac{\left(b^{2}-2b+2\right)\left(c-1\right)}{{\left(b^{2}-2\right)}^{2}}<0$, $\frac{\partial k^{NS}}{\partial c}=\frac{1-b}{b^{2}-2}<0$, we can infer that the scope of application for capacity sharing strategy will decrease with the increases of *b* and *c*. Although both strategies will be influenced by the increase of product cost *c*. The impact on capacity sharing system will be much more significant than that on capacity investment because $\frac{\partial k^{NS}}{\partial c}-\frac{\partial k^{IN}}{\partial c}=\frac{b}{4-2b^{2}}>0$.

**Proposition** **5.** (1) For M1, sharing environment-friendly capacity can reduce the loss caused by the limitation in capacity, but cannot make up all the loss, i.e., $\pi_{1}^{NN*}<\pi_{1}^{NS*}<\pi_{1}^{BM*}$. (2) M2 will also benefit from selling environment-friendly capacity to M1, i.e., $\pi_{2}^{BM*}<\pi_{2}^{NN*}<\pi_{2}^{NS*}$. (3) Facing with limitation in environment-friendly capacity, the capacity sharing strategy benefits both the capacity buyer and seller.

The conclusion is similar to that shown in Proposition 3. The difference is that M2 can benefit from M1’s capacity sharing decision because it can earn from selling capacity to M1. In other words, sharing the environment-friendly capacity brings a win-win situation. Both parties can benefit from the reallocation of the capacities. M2 is willing to share its environment-friendly capacity with M1 to promote the utilization of environment-friendly capacity.

Despite this, not all manufacturers can choose capacity sharing or investment, especially for those manufacturers with relatively large capacities *k*. Therefore, it is necessary to further discuss the question that which strategy does the manufacturer with limited environment-friendly capacity prefer.

## 4. Comparison between Capacity Investment and Capacity Sharing

### 4.1. The Scope of Application

We first compare the scope of application of these two strategies. Propositions 2 and 4 present applicable conditions for the adoption of capacity investment and sharing strategies. If only one strategy is feasible, the choice can be made easily. Comparing with the thresholds $k^{NS}$ and $k^{IN}$, we have $k^{NS}-k^{IN}=\frac{b(1-c)-2r}{2(b^{2}-2)}$. If $r>\frac{b(1-c)}{2}$, we have $k^{NS}>k^{IN}$ and the capacity sharing strategy holds a larger scope of application than capacity investment. With $k\in (k^{IN},k^{NS})$, only capacity sharing is feasible. On the contrary, if $\frac{b^{2}\left(1-c\right)}{2(b+2)}<r<\frac{b(1-c)}{2}$, capacity investment holds a larger scope of application. With $k\in (k^{NS},k^{IN})$, only capacity investment is feasible. In conclusion, we have:

**Proposition** **6.** *Given $\underline{r}<r<\bar{r}$,*

 *(1)* *Only strategy of sharing environment-friendly capacity is feasible if $k^{IN}<k<k^{NS}$ and $r>\frac{b(1-c)}{2}$;*
 *(2)* *Only strategy of investing environment-friendly capacity is feasible if $k^{NS}<k<k^{IN}$ and $r<\frac{b(1-c)}{2}$;*
 *(3)* *Both strategies are feasible if $k<\min\{k^{IN},k^{NS}\}$.*

**Figure 1:**
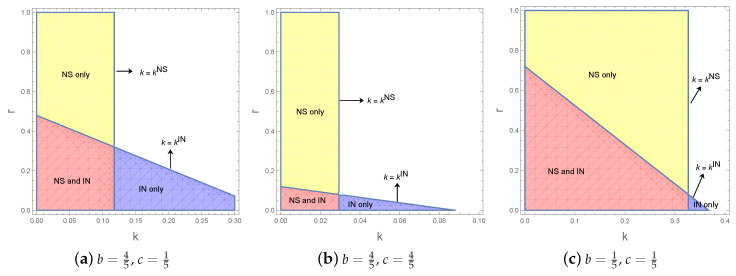
The feasible regions.

Although the relationship between the thresholds $k^{IN}$ and $k^{NS}$ is determined by other parameters, Proposition 6 clearly shows the conditions under which the two strategies can be used. Given values of *b* and *c*, we have Figure 1 to show the feasible regions of these two strategies with respect to *k* and *r*.

Figure 1 illustrates that the main factors determining the two strategies’ application scopes are the capacity *k* and cost coefficient *r*. Specifically, if $k<k^{NS}$, the capacity sharing strategy is feasible. When $k<k^{IN}$ and $r>\underline{r}$, capacity investment strategy is feasible. As $r<\bar{r}$ is equivalent to $k<k^{IN}$, we can represent the feasible region of both two strategies as $r\in (\underline{r},\bar{r})$. The feasible regions in Figure 1 demonstrated the conclusion in Proposition 6.

The adjustments of the values of *k* and *r* change the feasible regions of the two strategies, as shown in Figure 1a–c. From Figure 1a,b, we can see that the feasible regions of both strategies decrease with the increase of the cost *c*. Comparing the sizes of the feasible regions, it implies that the manufacturers with higher production costs are more likely to choose capacity sharing because the ones investing capacity will cause more production costs. From Figure 1a–c, we find that the feasible regions of both strategies increase with the decrease of the substitution coefficient *b*. It means that if the substitution between the competing productions is relatively strong, there is a strong possibility that both strategies will simultaneously fail to address the problem of a capacity limitation. The reason is that a substantial substitution leads to fierce competition, which weakens the willingness to cooperate.

### 4.2. The Profitability Analysis

We define such a set *S* to represent the case in which both capacity sharing and investment are feasible.

$$S=\left\{r,k|\underline{r}<r<\bar{r},\;k<k^{NS}\right\}\;or\;\left\{r,k|0<k<\min\left\{k^{IN},k^{NS}\right\},\;r>\underline{r}\right\}$$

For M1 with r,k∉S, at most one strategy can be used to improve profit, and then M1 can only choose the feasible one. For those with r,k∈S, two strategies are feasible, M1 will choose the one who brings it higher profits. In this section, we compare the profitability performances of capacity sharing and investment considering that k,r∈S, as shown in Region ABCD in Figure 1a.

**Proposition** **7.** *Comparing the profits obtained under IN and NS scenarios with k,r∈S, we have:*

 *(1)* *M1 will get more profits under NS scenario when the unit investment cost of environment-friendly capacity is relatively high; otherwise, it will get more profits under IN scenario, i.e.,*
  $$\left\{\begin{array}{c} \pi_{1}^{IN*}>\pi_{1}^{NS*}\;when\;\underline{r}<r<r_{1}\\ \pi_{1}^{IN*}\leq \pi_{1}^{NS*}\;when\;r_{1}\leq r<\bar{r} \end{array}\right.;$$
 *(2)* M2 will always get higher profits under NS scenario than IN scenario, i.e., $\pi_{2}^{IN*}<\pi_{2}^{NS*}$;
 *(3)* *Win-win situation exists when $r_{1}<r<\bar{r}$.*

If M1 has two choices, it will choose capacity investment when the unit investment cost on environment-friendly capacity *r* is relatively low; otherwise, it will choose capacity sharing. For M2, it prefers capacity sharing strategy because of the higher profits gaining in capacity sharing business. Of course, the choice of strategy is made by M1, M2 will always accept (to share capacity with M1).

**Figure 2:**
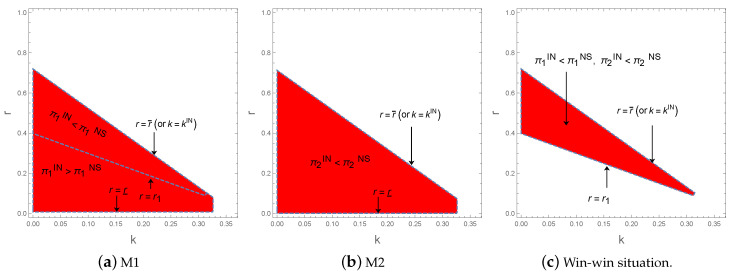
The profitability comparison.

Following the set $b=\frac{1}{5}$, $c=\frac{1}{5}$ of Figure 1c, we can draw Figure 2 to illustrate the Proposition 7.

From Figure 2a we can see a clear line dividing the region ABCD into two parts. This line is $r=r_{1}$. If $r<r_{1}$, that is the region below the line, we have $\pi_{1}^{IN*}>\pi_{1}^{NS*}$. Another region over the line represents $r>r_{1}$ and $\pi_{1}^{IN*}<\pi_{1}^{NS*}$. Figure 2b shows that M2 always prefers capacity sharing strategy rather than M1’s capacity investment strategy. As a result, the region above the line $r=r_{1}$ represents the win-win situation, in which both M1 and M2 can get more profits in capacity sharing strategy, as shown in Figure 2c.

## 5. Extension: The Stability Analysis of the Capacity Investment Strategy

Many local governments offer subsidies to firms for sustainable development, resource use, and energy efficiency [33]. For example, governments may provide subsidies to improve the development of low-carbon supply chain [34,35]. Hybrid Electric Vehicles are also subsidized to replace the fuel vehicles gradually [36,37,38]. As the investment in environment-friendly equipment or capacity may cost too much, the short-sighted manufacturers may produce with energy-intensive or highly-polluting manufacturing capacity. To avoid this, the government can subsidize the manufacturer to encourage it to upgrade its equipment to meet the requirements of environmentally friendly production. Besides, the subsidies may also take various forms. The three major ways the firms benefit from the subsidies include per-unit production subsidy [34], innovation effort subsidy [33] and capacity investment subsidy [39,40].

Based on the analysis in Proposition 2, the unit cost of capacity may prevent some of the manufacturers from investing capacities. Therefore, we consider the government to offer capacity subsidy to cover some of the unit cost, and ensure more and more manufacturers with $k>\frac{(b-2)(1-c)+2r}{2\left(b^{2}-2\right)}$ to invest capacities. If the subsidy is high enough, all the manufacturers with limited capacities will be willing to invest capacities.

Given the subsidy ρ for unit capacity investment, the profit function changes to be:

(11) $$\pi_{1}=\left(p_{1}-c\right)\left(k+\Delta k\right)-r\Delta k+\rho \Delta k$$

Based on Proposition 2, the condition for capacity investments changes to be $k<\min\{k^{\prime },\frac{b-bc+2(-1+c+r-\rho )}{2(-2+b^{2})}\}$. Making $k^{IN^{\prime }}=\frac{b(-c)+b+2(c-\rho +r-1)}{2(b^{2}-2)}$, all the manufacturers will invest capacities if $k^{IN^{\prime }}\geq k^{\prime }$. As the over-subsidization may not only waste government funds, but also induce the over-investment in capacity, the optimal subsidy should make $k^{IN^{\prime }}=k^{\prime }$. Then we have the following proposition.

**Proposition** **8.** Due to the high cost of the environment-friendly equipments, some firms may be not willing to invest in these equipments, which is harmful to the environment. The government should offer the subsidy $\rho^{*}=\frac{-b^{2}\left(1-c\right)+4r+2br}{2(2+b)}$ to ensure the limited-capacity manufacturer to invest capacity.

Interestingly, we find that the subsidy is not affected by the capacity of *k*. It implies that the government can successfully inspire manufacturers to invest environment-friendly capacities by providing subsidy $\rho^{*}$ for unit capacity investment, regardless of the initial capacity that the manufacturer.

Due to the high sunk cost of the environment-friendly capacity investment, the limited-capacity manufacturer will be very cautious in making decisions about its capacity investment. In this section, we consider M1 to be bounded rational and adjust the environment-friendly capacity investment in each period until it reaches the equilibrium. In reality, most of the firms make the evolutionary game due to the imperfect information during the decision-making process. As a result, it will try to use more complex expectations such as bounded rationality Bischi et al. [41], by which it endeavors to use local information based on the marginal profit Agiza and Elsadany [42]. To get the decisions at the period t+1, each will increase (decrease) the decisions at the period *t* if the marginal profit at the period *t* is positive (negative). As the first mover, the limited-capacity M1 makes decisions under incomplete information. Therefore, we consider that the capacity investment adjustment process follows:

(12) $$\Delta k\left(t+1\right)=\Delta k\left(t\right)+\alpha \cdot \Delta k\left(t\right)\cdot \frac{\partial \pi_{1}\left(t\right)}{\partial \Delta k(t)}$$

If $\frac{\partial \pi_{1}}{\partial \Delta k}<0$, Δk should be reduced for higher profit; if $\frac{\partial \pi_{1}}{\partial \Delta k}>0$, Δk should be added. α represents the adjustment speed. A higher α makes the system to reach the equilibrium more quickly. When the investment in environment-friendly capacity is adjusted to making $\frac{\partial \pi_{1}}{\partial \Delta k}=0$, the adjustment is over and then we have Δk(t+1)=Δk(t).

To solve this problem, we make Δk(t+1)=Δk(t) and then get two equilibrium outcomes $\Delta k_{1}^{*}=0$ and $\Delta k_{2}^{*}=-\frac{-1+c+(2+b)k}{2+b}$. Only $\Delta k_{2}^{*}$ is meaningful. Based on Jury criterion, the system can be stable when |J|<1, where,

(13) $$J=\frac{\partial \Delta k(t+1)}{\partial \Delta k(t)}=\frac{2+b^{3}\alpha \left(k+2\Delta k\right)+\left(b^{2}-2\alpha \right)\alpha \left(-1+c+2k+4\Delta k\right)+b\left(1-2k\alpha -4\alpha \Delta k\right)}{2+b}.$$

Substituting the meaningful equilibrium outcome $\Delta k_{2}^{*}$ into the Equation (13), we have $J=1+\frac{\left(2-b^{2}\right)\left(-1+c+2k+bk\right)\alpha }{2+b}$. Checking |J|<1, we can get the condition for the system stability.

**Proposition** **9.** *The adjustment speed should satisfy $0<\alpha <\frac{2(2+b)}{\left(-2+b^{2}\right)\left(-1+c+\left(2+b\right)k\right)}$ to guarantee the equilibrium of the decision-making system to be evolutionarily stable, so that the environment-friendly manufacturing system can be stable and sustainable.*

It is not difficult to prove that the upper bound of α is larger than zero. That is to say, if the limited-capacity manufacturer chooses to invest in the environment-friendly capacity, it can keep the system stable if it is patient enough and controls the adjustment speed at a lower level. From Proposition 9 we can also infer the stability condition w.r.t *b*, *c* and *k* and find the thresholds of them.

**Figure 3:**
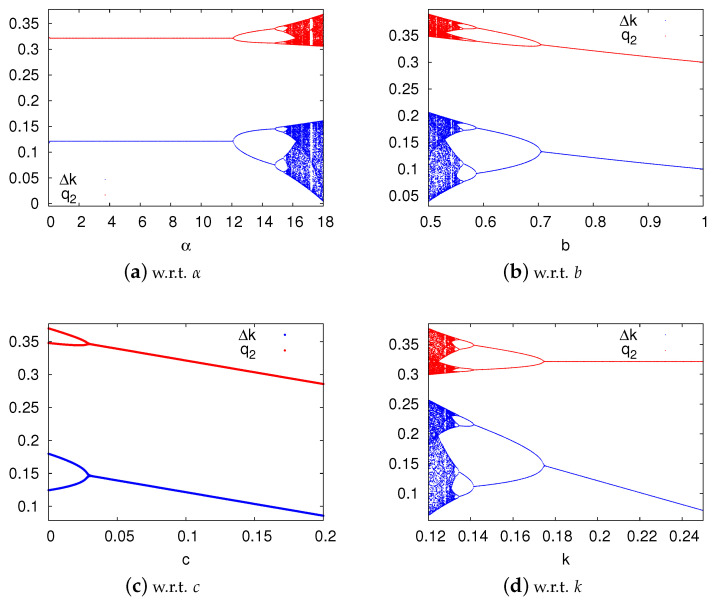
The bifurcation diagrams w.r.t. α, *b*, *c* and *k*.

Setting b=0.8, r=0.05, c=0.1, k=0.2, we can get the upper bound of α is 12.1107. The explanation of data selection and calculation is given in Appendix C. If α<12.1107 the system can be stable. Otherwise, the system will enter into chaotic state through period-doubling bifurcation, as shown in Figure 3a. Assign default value 10 for α, we can draw bifurcation diagrams w.r.t *b*, *c* and *k*.

**Figure 4:**
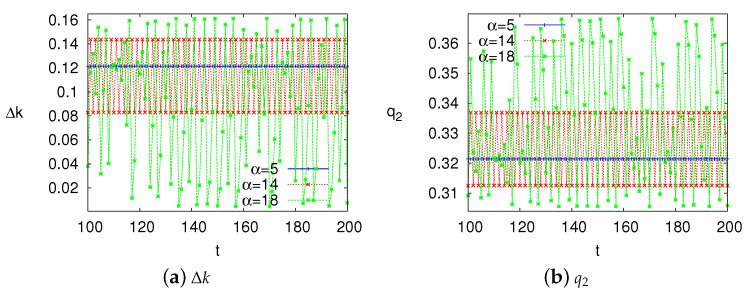
Time series of decisions under different system states.

In order to highlight the differences between stable and unstable systems, we assign different values for the parameters to simulate the different states of the system. Since Figure 3a shows the changes of system state w.r.t α, we can get the state of the system in a certain range of α. For example, the system is stable when α<12.1, and the system is the chaotic state when α>16.3. Therefore, we set α=5, α=14 and α=18 to simulate stable, period-doubling bifurcation and chaotic systems respectively. Figure 4 shows the time series of decision variables Δk and $q_{2}$ under stable, period-doubling bifurcation and chaotic systems. We can see that only in a stable system can the manufacturers have unique optimal decisions.

**Figure 5:**
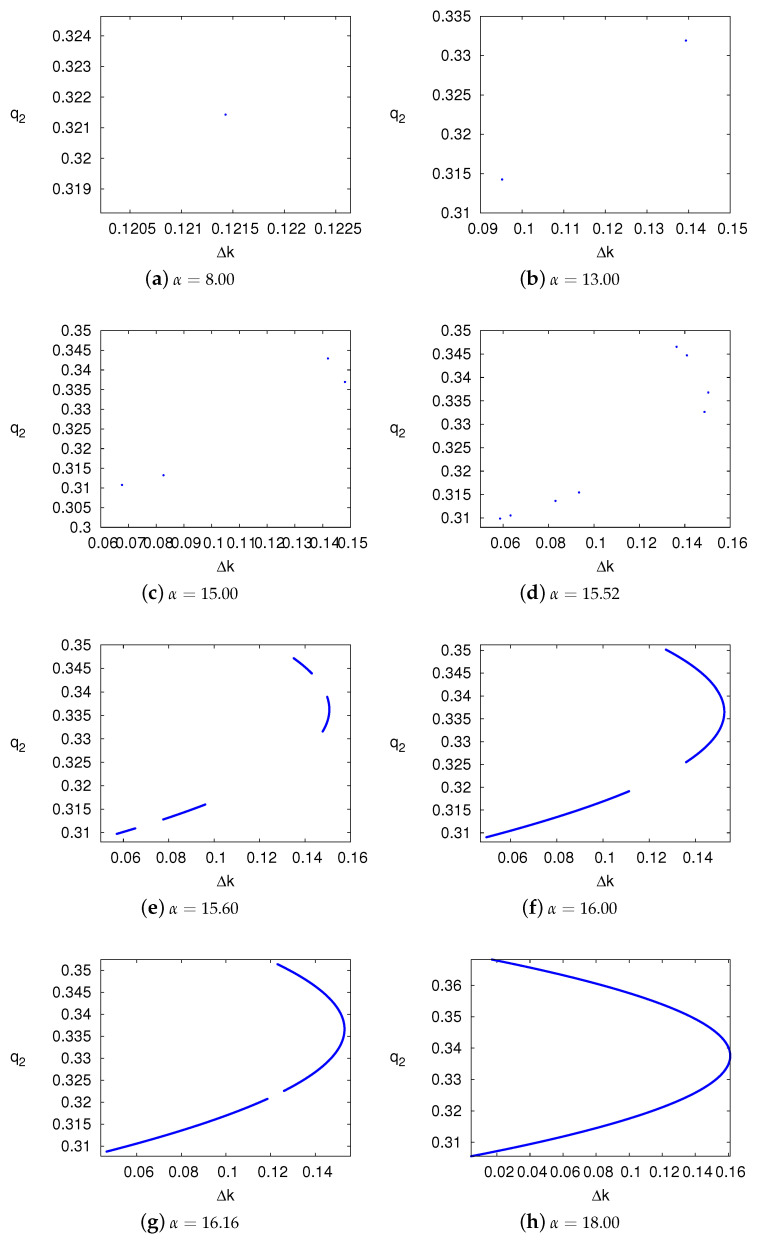
The chaotic attractors w.r.t α.

Figure 5 clearly shows how a stable system enters into a chaotic state from period-doubling bifurcation paths. Even in an unstable system, the decision variables’ iterative trajectories can be quite different with the change of other parameters. From a macro perspective, the iterative trajectories appear to be regular. However, if we magnify the pictures, we can find the chaotic points forming the iterative trajectories. This irregular order is one of the most essential characteristics of the chaotic system. To keep the system stable, the adjustment speed should be lower than its threshold, while *b*, *c* and *k* should be larger than their thresholds.

## 6. Management Implications

Under stiffening environmental regulations in many countries, environmental management has been a strategic imperative for manufacturers. A large number of manufacturers, especially small and micro companies, still employed the outdated production and processing techniques, causing great pollution issues and threatening the health of local residents. The analytical results have shown that the limitation of environment-friendly capacity will cause distress to their development. Besides fully utilizing the capacities which meet the environmental standards, updating the manufacturing capacity or transforming to the green production mode are essential to their survival, since it is proved that both strategies can reduce the loss caused by capacity limitation.

The manufacturer should choose the capacity investment strategy if the capacity investment cost is relatively low. Since the former section has shown that the government can use the subsidy to successfully motivate the manufacturer to invest in the environmental-friendly capacity in spite of their initial capacities, the government plays a crucial role in guiding the development of environmental sustainability in the manufacturing industry. For manufacturers who intend to invest in environment-friendly capacity, it is advisable to seize the opportunity of getting subsidy from the government. If the manufacturer has a long-term investment plan in green manufacturing, it should adjust the volume of capacity investment at a relatively low speed to keep the supply chain system stable. When they build sufficient environment-friendly capacity, they can also share the capacity with other manufacturers to cover their investment and gain more profits.

Compared with the capacity investment strategy, it is the capacity sharing strategy that can benefit both manufacturers with the limited capacity and the manufacturer with sufficient capacity. When the capacity investment cost satisfies a certain condition, the win-win situation exists. For the manufacturer faces a high financial burden in investment, it is wise to adopt the pattern of capacity sharing, seeking to join a shared factory with existing capacity, techniques and labor. Capacity sharing will bring great economic benefit and environmental improvements. For example, in the Zhongwei air-conditioning shared factory we mentioned in the Introduction, the previous polluting manufacturers can use the standardized workshop in a shared factory, and realize the clean production in the whole process. With the capacity sharing mode in the air-conditioning industry of the Wucheng County, the tax revenue from air-conditioning manufacturers raised by 35.05%. (http://news.eastday.com/eastday/13news/auto/news/china/20180510/u7ai7692988.html).

The fact that sharing the environment-friendly capacity inspires the manufacturers to form an alliance of mutual benefits has already got a large amount of actual confirmation in the manufacturing industry. For example, in foundry production, Hua Xin Co.,Ltd innovates the “short” casting process to save energy and reduce emissions. For 200,000 tons of casting, the “short” casting process can save 48,000 tons of standard coal per year and reduce 52,000 tons of carbon dioxide. Considering plenty of idle electric smelting furnaces and flexible production equipment is unaffordable for small and medium manufacturers that need them, this company proposed a shared smelting center to lease the equipment and charge by the quantity of processed products. Profits of participants can be increased by 3–5% (https://new.qq.com/omn/20180218/20180218A04YYM.html). In the textile manufacturing industry, Companion Group achieved green production with the use of automatic dyeing technology and equipment, and built shared factory to share the printing equipment and techniques. Compared with the traditional printing and dyeing processing mode, the new mode can improve production efficiency by 28%, reduce sewage discharge by 68%, and reduce overall energy consumption by 45% (http://www.taweekly.com/zx/xygz/201904/t20190429_3869451.html).

## 7. Conclusions

This paper discusses two strategies that the manufacturer with limited environment-friendly capacity can choose to reach green manfuacturing, i.e., capacity investment in establishing environment-friendly capacity and capacity sharing with a manufacturer with sufficient capacity. Considering the existence of competition between two manufacturers, we build game theoretical models for the supply chain under the cases of no capacity investment or sharing, capacity investment, and capacity sharing. Based on the equilibrium results in each case, we present the application scopes of each strategy and provide the conditions when both two strategies are feasible. Through a comprehensive comparison on the profitabilities of manufacturers, we get each manufacturer’s preference on these two strategies. Furthermore, we use chaos theory to analyze the nonlinear characteristics of the system and put forward conditions to keep the system stable, considering the manufacturer with limited capacity can gradually adjust its capacity investment.

The results show that the capacity limitation and capacity overinvestment will be detrimental to the manufacturer with limited capacity. Both appropriate capacity investment and capacity sharing can effectively reduce the profit loss of the manufacturer caused by insufficient capacity. The manufacturer’s capacity investment is unfavorable to the manufacturer with sufficient capacity while capacity sharing will be beneficial to him. The application scope of these two strategies depends on the initial environment-friendly capacity and the unit cost of investment in environment-friendly capacity. When the unit capacity investment cost is within a specific range, both strategies are feasible. When the cost of investment is relatively low, capacity investment is an effective way to reach green manufacturing. Besides, the subsidy of the government can encourage the manufacturer in investing environment-friendly capacity. When the manufacturer makes a capacity investment, the adjustment speed should be in a proper range. When the cost coefficient is relatively high, the capacity sharing is a more preferable way for the limited-capacity manufacturer and a win-win situation exists for both manufacturers. As this paper considers the competition between the capacity buyer and seller, it also indicates that the competitors can cooperate with each other in sharing environment-friendly capacities. By investigating the co-opetition game model, this paper studies the resource allocation between the competition side and the cooperation side. The findings can provide managerial insights for manufacturers in strategy selection on environment-friendly capacity investment or sharing, and provide a way to balance the profitability performance and stability performance for the decision-makers.

In this paper, we do not consider the differences in the manufacturing process between two manufacturers. As the production process may lead to different costs, we will further expand the analysis considering the differences in production costs between the capacity buyer and seller and study its influence on the choice of capacity sharing and capacity investment.

## Appendix A. The Equilibrium Profits

$$\begin{array}{c} {\pi_{1}}^{BM*}=\frac{{\left(-1+c\right)}^{2}}{{\left(2+b\right)}^{2}},{\pi_{2}}^{BM*}=\frac{{\left(-1+c\right)}^{2}}{{\left(2+b\right)}^{2}},\\ {\pi_{1}}^{NN*}=\frac{1}{2}k\left(b\left(-1+c\right)+b^{2}k-2\left(-1+c+k\right)\right),{\pi_{2}}^{NN*}=\frac{1}{4}{\left(-1+c+bk\right)}^{2},\\ {\pi_{1}}^{IN*}=-\frac{-4b\left(-1+c\right)\left(-1+c+r\right)+b^{2}\left(1-2c+c^{2}-8kr\right)+4\left(1+c^{2}+2c\left(-1+r\right)-2r+4kr+r^{2}\right)}{8(-2+b^{2})},\\ {\pi_{2}}^{IN*}=\frac{{\left(4+b^{2}\left(-1+c\right)-4c+2b\left(-1+c+r\right)\right)}^{2}}{16{\left(-2+b^{2}\right)}^{2}},\\ {\pi_{1}}^{NS*}=\frac{\left(\begin{array}{c} -16b\left(-1+c\right)\left(-1+c-2k\right)-16b^{3}\left(-1+c\right)k+3b^{5}\left(-1+c\right)k\\ +3b^{6}k^{2}-6b^{4}k\left(-2+2c+5k\right)+8(1+c^{2}+12k-12k^{2}-\\ 2c\left(1+6k\right))+8b^{2}\left(1+c^{2}-8k+12k^{2}+c\left(-2+8k\right)\right) \end{array}\right)}{2{\left(8-3b^{2}\right)}^{2}},\\ {\pi_{2}}^{NS*}=\frac{\left(\begin{array}{c} \left(-2+b\right)(b^{3}k^{2}+2b^{2}k\left(-1+c+k\right)+b(1+c^{2}+4k-4k^{2}\\ -2c\left(1+2k\right))-2\left(3+3c^{2}-4k+4k^{2}+c\left(-6+4k\right)\right)) \end{array}\right)}{4(8-3b^{2})}. \end{array}$$

## Appendix B. The Proofs

**Proof of Lemma 1.** Using Lagrange multiplier method, problem (1) can be modeled as

$$\max\pi_{1}=\left(p_{1}-c\right)q_{1}+\lambda \left(q_{1}-k\right)$$

If the capacity is sufficient, the condition $q_{1}\leq k$ is relaxed and λ=0. According to $\frac{d^{2}\pi_{i}}{d{q_{i}}^{2}}=-2<0$ the optimal solutions can be solved by the first order condition $\frac{d\pi_{i}}{dq_{i}}=0$. If the capacity is insufficient, the condition $q_{1}\leq k$ works. Then we have $q_{1}=k$ and the optimal solutions can be solved by the first order condition $\frac{d\pi_{1}}{d\lambda }=\frac{d\pi_{2}}{dq_{2}}=0$. □

**Proof of Proposition 1.** With ${\pi_{1}}^{BM*}=\frac{{\left(-1+c\right)}^{2}}{{\left(2+b\right)}^{2}}$, ${\pi_{1}}^{NN*}=\frac{1}{2}k\left(b\left(-1+c\right)+b^{2}k-2\left(-1+c+k\right)\right)$, we can solve out that the condition under which ${\pi_{1}}^{BM*}>{\pi_{1}}^{NN*}$ is that $0<k<\frac{1-c}{2+b}\left|\right|k>\frac{2(-1+c)}{-4-2b+2b^{2}+b^{3}}$. As $0<k<\frac{1-c}{2+b}$ is the basic condition for the limited capacity case, we have ${\pi_{1}}^{BM*}>{\pi_{1}}^{NN*}$ is always true. Similarly, with ${\pi_{2}}^{BM*}=\frac{{\left(-1+c\right)}^{2}}{{\left(2+b\right)}^{2}}$, ${\pi_{2}}^{NN*}=\frac{1}{4}{\left(-1+c+bk\right)}^{2}$, we have ${\pi_{2}}^{BM*}<{\pi_{2}}^{NN*}$ if $0<k<\frac{1-c}{2+b}\left|\right|k>\frac{2(-1+c)}{-4-2b+2b^{2}+b^{3}}$. It means that ${\pi_{2}}^{BM*}<{\pi_{2}}^{NN*}$ is always true. □

**Proof of Lemma 2.** The problem solving process has been placed in the main text. □

**Proof of Proposition 2.** As Δk>0 guarantees M1 to invest capacity, we solve the inequality Δk>0 w.r.t. *k* and get $k<\frac{b-bc+2(-1+c+r)}{2(-2+b^{2})}$ and $-\frac{b^{2}\left(-1+c\right)}{2(2+b)}<r<\frac{1}{2}\left(-2+b\right)\left(-1+c\right)$. □

**Proof of Proposition 3.** The orders can be got by comparing the analytical results shown in Appendix A. □

**Proof of Lemma 3.** The problem solving process has been placed in the main text. □

**Proof of Proposition 4.** The results can be obtained by solving the inequality $q_{t}^{NS*}>0$. □

**Proof of Proposition 5.** The orders can be got by comparing the analytical results shown in Appendix A. □

**Proof of Proposition 6.** Given the profits in Appendix A, we can making differences between the analytical profits obtained in any two scenarios. □

**Proof of Proposition 7.** Given the profits $\pi_{i}^{IN*}$ and $\pi_{i}^{NS*}$, we can compare them by Mathematica software. Under the condition $r>-\frac{b^{2}\left(-1+c\right)}{2(2+b)},0<k<\min\left\{\frac{-1+b+c-bc}{-2+b^{2}},\frac{b-bc+2(-1+c+r)}{2(-2+b^{2})}\right\}$, we try to solve the range of *r* and making the *r* locating in this range to satisfy $\pi_{2}^{IN*}>\pi_{2}^{NS*}$. The output is “False”. It means that the basic set $r>-\frac{b^{2}\left(-1+c\right)}{2(2+b)},0<k<\min\left\{\frac{-1+b+c-bc}{-2+b^{2}},\frac{b-bc+2(-1+c+r)}{2(-2+b^{2})}\right\}$ is a subset of the set of *r* representing $\pi_{2}^{IN*}<\pi_{2}^{NS*}$. Every *r* locating in the basic set will locate in the set making $\pi_{2}^{IN*}<\pi_{2}^{NS*}$. In the same way, we solve out the range representing $\pi_{1}^{IN*}>\pi_{1}^{NS*}$, that is $\underline{r}<r<r_{1}$, where $r_{1}=1+\frac{1}{2}b\left(-1+c\right)-c-2k+b^{2}k+\Phi \left(b^{2}-2\right)$ and $\Phi =\sqrt{\frac{32b^{3}\left(1-c\right)k+6b^{5}\left(-1+c\right)k+6b^{6}k^{2}+16b\left(1-c\right)\left(1-c-2k\right)-8{\left(1-c-2k\right)}^{2}-6b^{4}k\left(1-c-6k\right)-8b^{2}\left(1-2c+c^{2}+4k-4ck-8k^{2}\right)}{{\left(8-3b^{2}\right)}^{2}\left(-2+b^{2}\right)}}$. □

**Proof of Proposition 8.** With subsidy ρ, the optimal investment is

$$\Delta k=\frac{b-bc-2b^{2}k+2\left(-1+c+2k+r-\rho \right)}{2(-2+b^{2})}$$

Making Δk≥0, we have

$$k<\frac{b-bc+2(-1+c+r-\rho )}{2(-2+b^{2})}.$$

When $\frac{b-bc+2(-1+c+r-\rho )}{2(-2+b^{2})}\geq \frac{1-c}{2+b}$, we know that all limited-capacity manufacturers will invest capacities. The result is

$$\rho \geq \frac{b^{2}\left(-1+c\right)+4r+2br}{2(2+b)}.$$

Over subsidies may lead to negative effects, including the overinvestment. The optimal subsidy is

$$\rho^{*}=\frac{b^{2}\left(-1+c\right)+4r+2br}{2(2+b)}$$

 □

**Proof of Proposition 9.** With $J=1+\frac{\left(2-b^{2}\right)\left(-1+c+2k+bk\right)\alpha }{2+b}$, we can get the condition for system stability by solving |J|<1. □

## Appendix C. The Explanation of Data Selection

Setting b=0.8, to guarantee the basic condition $\bar{c}>0$, we solve the inequality $\frac{b^{2}-4r-2br}{b^{2}}>0$ and get r<0.1143. Then we set r=0.05 and get the basic condition $\bar{c}=0.5625$. Then we set c=0.1, we can get $k<\frac{1-c}{b+2}=0.3214$. So we set k=0.2 and get the upper bound of $\alpha =\frac{2(2+b)}{\left(-2+b^{2}\right)\left(-1+c+\left(2+b\right)k\right)}\;=\;12.1107$.
